# Current wishes to die; characteristics of middle-aged and older Dutch adults who are ready to give up on life: a cross-sectional study

**DOI:** 10.1186/s12910-021-00632-4

**Published:** 2021-05-21

**Authors:** Roosmarijne M. K. Kox, H. Roeline W. Pasman, Martijn Huisman, Wim Benneker, Bregje D. Onwuteaka-Philipsen

**Affiliations:** 1 Department of Public and Occupational Health, Amsterdam Public Health Research Institute, Amsterdam UMC, University of Amsterdam, P.O. Box 7057, 1007 MB Amsterdam, Netherlands; 2 Department of Epidemiology and Biostatistics, Amsterdam Public Health Research Institute, Amsterdam UMC - Location VU University Medical Center, Amsterdam, The Netherlands; 3 Department of Sociology, Vrije Universiteit Amsterdam, Amsterdam, The Netherlands; 4 Department of General Practice and Elderly Care Medicine, University Medical Center Groningen, University of Groningen, Groningen, The Netherlands

**Keywords:** Wish to die, Death thoughts, Middle-aged and older adults, Completed life, Political debate, The Netherlands

## Abstract

**Background:**

Literature shows that middle-aged and older adults sometimes experience a wish to die. Reasons for these wishes may be complex and involve multiple factors. One important question is to what extent people with a wish to die have medically classifiable conditions.

**Aim:**

(1) Estimate the prevalence of a current wish to die among middle-aged and older adults in The Netherlands; (2) explore which factors within domains of vulnerability (physical, cognitive, social and psychological) are associated with a current wish to die; (3) assess how many middle-aged and older adults with a current wish to die do not have a medically classifiable condition and/or an accumulation of age-related health problems.

**Methods:**

Data of 2015/16 from the Longitudinal Aging Study Amsterdam were used for this cross-sectional study (1563 Dutch middle-aged and older adults aged between 57 and 99 years), obtained through structured medical interviews and self-reported questionnaires. Three experienced physicians assessed whether the participants with a current wish to die could be classified as having a medically classifiable condition and/or an accumulation of age-related health problems.

**Results:**

N = 62 participants (4.0%) had a current wish to die. Having a current wish to die was associated with multiple characteristics across four domains of vulnerability, among which: self-perceived health, problems with memory, self-perceived quality of life and meaningfulness of life. Fifty-four participants with a current wish to die were assessed with having a medically classifiable condition, of which one was also assessed with having an accumulation of age-related health problems. Six people were assessed to have neither, and for two people it was unclear.

**Conclusion:**

A small minority of middle-aged and older adults in the Netherlands have a current wish to die. Most of them can be classified with a medical condition and one person with an accumulation of age-related health problems. Furthermore, the findings show that having a current wish to die is multi-faceted. There is still a need for more knowledge, such as insight in to what extent suffering stemming from the medical classifiable disease contributes to the development of the wish to die.

**Supplementary Information:**

The online version contains supplementary material available at 10.1186/s12910-021-00632-4.

## Background

The experience of wishes to die among older adults has been studied before worldwide and prevalence estimates ranged between 4.6 and 9.5% [1–5]. In the Netherlands, two studies including also middle-aged adults have been performed. A study in 2005 using data of the fifth wave of Longitudinal Aging Study Amsterdam (LASA) found that of a representative group of Dutch middle-aged and older adults aged between 58 and 98 years 3.4% had a current wish to die [6]. A more recent Dutch study, specifically looking at a persistent death wish in the absence of severe illness, reported a prevalence of 1.25% among middle-aged and older adults aged 55 years and older [7]. More than half of these people had an active wish to die [7]. Van Wijngaarden et al. [7] reported that almost 90% of middle-aged and older adults with an active wish to die considered ending their live in the past year and a minority even made plans regarding their death wish. Dutch qualitative studies pointed out that participants with a strong wish to die often had no plans to end their lives in the short term [8, 9]. Moreover, participants also mentioned having reasons to live besides having a wish to die, for example having (grand)children and being part of their lives [8].

It is known that wishes to die among middle-aged and older adults are associated with several vulnerability factors such as loneliness, age, medical status, depression, self-reported health, a small social network, falls, and serious financial problems [1, 3–7, 10, 11]. Empirical studies that describe the characteristics of people with wishes to die are important for improving understanding of the context in which they arise. Multiple domains of vulnerability can be distinguished in middle-aged and older adults: e.g. physical, cognitive, social and psychological vulnerability [12]. The aforementioned studies have identified factors associated with having a wish to die in many of these domains. However, most of these studies were able to include limited sets of factors, while it is known that death wishes in older adults develop in complex contexts and often implicate a broad range of factors [5]. It is currently unknown which factors are the most important within the four domains of vulnerability for having a wish to die.

Since the legalization of euthanasia and physician-assisted suicide (EAS) in the Netherlands in 2002, a social and ethical debate is ongoing concerning to what extent the current law can cover the situation of older adults with a so-called completed life [13], a state in which the value and meaning of life are reduced in such a way that the person will choose death over life [14]. A physician will not be prosecuted for performing EAS if all six criteria of due care (Box 1) are met and he/she reports the euthanasia to the Regional Euthanasia Review Committee [14]. The euthanasia act does not require a life-threatening condition or limited life expectation [14]. Therefore, besides terminally ill patients, EAS can also be received by patients with psychiatric disorders, dementia or an accumulation of age-related health problems (such as visual impairment, hearing impairment, osteoporosis, arthrosis, balance disorders, and cognitive decline) [14–16]. However, according to the case law and legislative history, unbearable suffering must stem from a medically classifiable (somatic or psychiatric) condition [14]. Older adults who experience a completed life are currently not eligible for euthanasia if their suffering does not stem from a medically classifiable condition [14]. Important in the debate about completed life is therefore how often it occurs that the feeling of a completed life is not related to a medically classifiable condition, such as age-related health problems [13]. Committee Schnabel, a Committee of Wise People introduced by the Dutch government to research the legal possibilities and the social dilemmas of EAS for people with an experienced completed life, expected the group of older adults who experience a completed life and without having a medically classifiable condition to be small [17]. Although several studies examined the wish to die among middle-aged and older adults, most of the studies did not make a distinction between healthy middle-aged and older adults and middle-aged and older adults who are (severely) ill [17, 18].
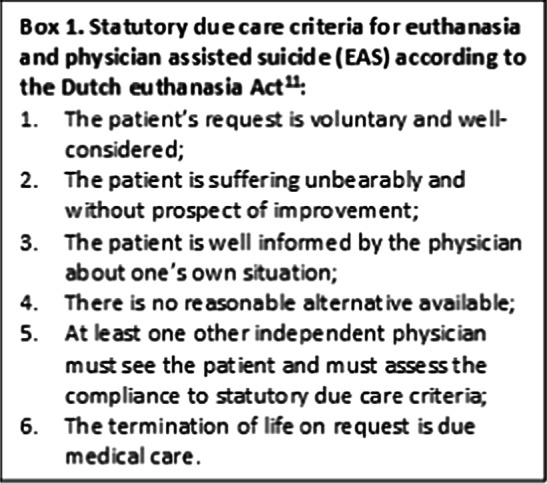


Therefore, this study explores the prevalence and characteristics of middle-aged and older adults aged between 57 and 99 years with a wish to die using data of the eighth wave of the LASA cohort, similar to the study in 2005[6]. We investigated three research questions: What percentage of middle-aged and older adults have a current wish to die? Which domains of vulnerability and what characteristics are associated with a current wish to die? And how many middle-aged and older adults with a current wish to die do not have a medically classifiable condition and/or an accumulation of age-related health problems?

## Methods

### Design

A cross-sectional study was conducted using data from the Longitudinal Aging Study Amsterdam (LASA). LASA studies the physical, emotional, cognitive, and social functioning of a nationally representative sample of middle-aged and older adults in the Netherlands. The sample was drawn from three culturally distinct geographic regions in the Netherlands (secular, protestant, and catholic), namely from the cities of Zwolle, Oss and Amsterdam and surrounding rural areas [19, 20]. In LASA, the oldest old and men were oversampled [19]. In 1992/93, baseline measurement took place among 3107 participants, aged between 55 and 85 years. Follow-up measurements of the LASA study have been conducted about every 3 years using different methods: general interviews, medical interviews and self-administered questionnaires [19, 20]. Sampling and data collection of the LASA study have been described in detail elsewhere [19].

This study is based on LASA data of 2015/16 (wave 8), response rate of 79.5%, where questions about wishes to die were included in the self-administered questionnaire [21]. An important reason for respondents to not fill in the questionnaire is inability; the questionnaire was more often not completed by people who are older and have more diseases [19].

### Sample

The sample for the current study consisted of 1608 middle-aged and older adults aged 57–99 years who completed the self-administered questionnaire. Forty-five participants were excluded from the analysis because of missing values on the dependent variable, hence the total study population consisted of 1563 participants aged ≥ 57. 53.6% of the total study population were female and the highest proportion had an age between 65 and 75 (41.5%).

### Measurement instruments

#### Dependent variable

To come to the dependent variable of a current wish to die, four questions in the questionnaire are relevant. Two questions, derived from the Paykel suicide scale: ‘Have you ever felt like life was not worth living?’ and ‘Have you ever wished you were dead, for instance, that you could go to sleep and not wake up?’ (yes; no; don’t know) [22]. Two other questions, derived from The Scale of Suicide Ideation (SSI), were about feelings towards living and feelings towards dying: ‘What were your feelings toward living/dying the past week? Did you wish to live/die, and how strong was this wish?’ (moderate to strong wish; weak wish; no wish) [23]. All four questions were combined into a dependent variable with five categories: (1) has never had death thoughts or wishes; (2) has at some point experienced death thoughts and/or wishes; (3) had only a weak wish to live in the past week, but no wish to die in the past week; (4) had no wish to live in the past week and/or a weak wish to die in the past week; (5) had a moderate to strong wish to die in the past week. For the analyses we merged the last three categories into one, because of a limited number of participants in three of the categories. This was possible as literature shows that middle-aged and older adults can experience a balance in feelings towards living and dying [8, 9]. Thus the categories of the dependent variable were: (1) has never had death thoughts or wishes; (2) has at some point experienced death thoughts and/or wishes; (3) and has a current wish to die or a weak wish to live (hereafter: current wish to die). An additional file shows the recoding of the four questions into the dependent variable (see Additional file 1). As mentioned before, data regarding wishes to die were collected through the self-administered questionnaire. However, all participants have had contact with interviewers of LASA, who are alert to signs of physical or psychological distress of participants and report when they believe that a participant needs to be checked, but only after participants give consent for doing so.

#### Independent variables

Various characteristics were included as independent variables [21]. These characteristics include demographics (sex, age, marital status, level of attained education, level of urbanization, housing) and characteristics that we categorized into four domains of vulnerability [12]: (1) physical vulnerability (number of chronic diseases, incontinence, pain, hearing and visual impairment, dizziness, number of activities with some difficulty or worse, balance, whether or not help was received with personal care, sufficiency of received help, health problems limit normal activities, self-perceived health), (2) cognitive vulnerability (MMSE score, problems with memory), (3) social vulnerability (loneliness, network size, health problems limit social activities, self-perceived quality of life, financial problems, conflict with other persons(s), illness of partner or spouse, death of sons, daughters and/or grandchildren) and (4) psychological vulnerability (depressive symptoms, depression past year, anxiety, self-esteem, perceived self-efficacy, mastery, lately satisfied with life and meaningfulness of life). An additional file shows all characteristics in more detail (see Additional file 2).

#### Medically classifiable condition and accumulation of age-related health problems

Three SCEN (Support and Consultation on Euthanasia in the Netherlands) physicians, of whom one was retired, were asked to assess which participants with a current wish to die did not have a medically classifiable condition. SCEN physicians are independent expert doctors who can be consulted by another physician for support when receiving an EAS request of a patient, and to assess the compliance to statutory due care criteria [14]. Besides being a SCEN physician, one of the approached physicians was an elderly care physician and the other two were general practitioners. A confidentiality statement was signed by all three SCEN physicians, to protect the privacy of LASA participants. All cases with a current wish to die were anonymously described regarding their physical and mental health, functional limitations, and self-perceived health, using information from the LASA measurements. The case descriptions were sent to the SCEN physicians either per mail or per post together with an explanation of the described characteristics regarding their measurement method. An additional file shows an example of a fictitious case description (see Additional file 3), for which parts of different cases were aggregated. Based on the information provided, the physicians independently assessed whether or not a person had one (or more) medically classifiable condition(s) and whether or not someone had an accumulation of age-related health problems. SCEN physicians were chosen, because they are used to making such assessments as part of their work. Differences in judgement between the three physicians were discussed in a meeting to gain consensus.

### Analysis

IBM SPSS version 22 (IBM Analytics) was used to carry out the statistical analyses. Descriptive statistics were used to describe the study population and the prevalence of a current wish to die among the study population. The prevalence estimates were weighted to the total Dutch population aged between 57 and 99 years (n = 4,726,351) [19]; all death wish prevalences per 5 years of age were multiplied with the prevalences of the total Dutch population per 5 years of age [24]. Bivariate analyses, using a chi square test and an analysis of variance (ANOVA), were performed with the three categories of the dependent variable to identify factors associated with a wish to die. Multivariate logistic regression analyses were done between the first group (has never had death thoughts or wishes) and the third group (has a current wish to die). All variables associated with a current wish to die (*p* value < 0.05) were entered in multivariable models per category (demographics; physical vulnerability; cognitive vulnerability; social vulnerability; psychological vulnerability), using a manual backward elimination with a *p* value of < 0.05. All models were corrected for age; correcting for sex as well had an negligible effect on the associations. Balance was analysed using only the first two categories (no balance disorder and balance disorder). In the multivariable analyses, depressive symptoms according to the CES-D score and depression according to the diagnostic CIDI interview were combined because of multicollinearity. This variable was categorized into three categories [25]. Descriptive statistics were also used to assess how many participants with a current wish to die did not suffer from a medically classifiable condition and/or an accumulation of age-related health problems.

## Results

### Prevalence of a current wish to die

Table 1 shows that about 84% of the participants never have had death thoughts or wishes and 12.1% of the participants reported that they have at some point experienced death thoughts and/or wishes, but they did not have a wish to die in the past week. The remaining 4% (weighted and unweighted) of the participants (n = 62) had a current wish to die or a weak wish to live. People with a current wish to die were on average more often female (64.5% compared to 52.3% and 58.7%) and more often aged 85 years or older (22.6% compared to 7.3% and 6.9%). The characteristics of the study sample are provided in Table 2.

**Table 1:** Unweighted and weighted prevalence of death thoughts and wishes to die

	n	Unweighted	Weighted
		%	%^a^
Has never had death thoughts or wishes	1312	83.9	83.1
Has at some point experienced death thoughts and/or wishes, but had no wish to die in the past week and a moderate to strong wish to live in the past week	189	12.1	13.0
Current wish to die or a weak wish to live	62	4.0	4.0
*Had only a weak wish to live in the past week, but no wish to die in the past week*	*15*	*1.0*	*1.0*
*Had no wish to live in the past week and/or a weak wish to die in the past week*	*36*	*2.3*	*2.2*
*Had a moderate to strong wish to die in the past week*^b^	*11*	*0.7*	*0.8*
Total	1563	100.0	100.1

^a^Weighted percentage for the total Dutch middle-aged and older population (n = 4,726,351)

^b^3 participants reported having no wish to live, 5 participants reported having a weak wish to live, and 3 participants reported having a moderate to strong wish to live in the past week

**Table 2:** Characteristics of middle-aged and older adults who have never had death thoughts or wishes, who have at some point experienced death thoughts or wishes and who have a current wish to die or a weak wish to live ordered per domain of vulnerability

Characteristics per domain of vulnerability	Total	Never death thoughts or wishes	Has had death thoughts or wishes	Current wish*	*p* value
	(n = 1563)	(n = 1312)	(n = 189)	(n = 62)	
	%/µ (SD)	%/µ (SD)	%/µ (SD)	%/µ (SD)	
*Demographics*					
Sex					0.053
Male	46.4	47.7	41.3	35.5	
Female	53.6	52.3	58.7	64.5	
Age^a^					< 0.001
< 65 years	27.6	26.4	38.1	21.0	
65 to < 75	41.5	42.5	37.0	33.9	
75 to < 85	23.1	23.9	18.0	22.6	
≥ 85 years	7.9	7.3	6.9	22.6	
Marital status^a^					< 0.001
Never married	7.4	6.0	16.9	8.1	
Married and registered partnership	67.2	70.7	51.9	41.9	
Divorced	8.1	6.9	13.8	14.5	
Widowhood	17.3	16.4	17.5	35.5	
Level of attained education					0.107
Low	32.8	33.7	24.9	38.7	
Middle	38.5	38.3	41.3	32.3	
High	28.7	28.0	33.9	29.0	
Level of urbanization^b^					0.029
Sparsely populated (< 1000)	44.9	46.4	38.8	33.9	
Densely populated (≥ 1000)	54.9	53.6	61.2	66.1	
Housing^c,d^					0.007
Independent living	99.4	99.5	99.5	95.2	
Dependent living (residential or nursing home	0.6	0.5	0.5	4.8	
*Physical vulnerability*					
Number of chronic diseases (out of 7)^e^					0.020
0 diseases	24.6	25.9	18.5	14.5	
1 disease	39.1	39.1	40.2	35.5	
≥ 2 diseases	36.3	35.0	41.3	50.0	
Incontinence^a,b^					0.027
No	94.0	96.2	93.5	90.3	
Yes	4.3	3.8	6.5	9.7	
Pain^b^					< 0.001
No pain [5]	67.2	71.9	53.6	41.9	
Some pain (6–7)	17.7	17.2	21.3	25.8	
Many pain (> 7)	13.2	10.9	25.1	32.3	
Hearing impairment^a,b^					0.011
No impairment	66.3	70.0	61.6	61.7	
Some difficulty	26.0	26.0	33.5	26.7	
Much difficulty/not able to hear	4.3	4.0	4.9	11.7	
Visual impairment^a,b,d^					0.294
No impairment	85.3	90.1	86.7	85.0	
Some difficulty	7.5	7.4	10.5	10.0	
Much difficulty/not able to see	2.5	2.5	2.8	5.0	
Dizziness^a,b^					< 0.001
No	87.4	91.9	84.3	75.0	
Yes	9.3	8.1	15.7	25.0	
Number of activities with some difficulty or worse (out of 7)^b^					< 0.001
0 activities	47.0	49.5	38.2	20.0	
1 activity	21.7	21.4	22.0	28.3	
≥ 2 activities	31.3	29.1	39.8	51.7	
Balance^a,b^					< 0.001
No balance disorder	84.8	89.0	83.6	72.7	
Balance disorder	10.4	9.6	14.8	25.5	
Refused/not able to	1.4	1.4	1.6	1.8	
Whether or not help was received with personal care^a^					0.023
No	96.8	97.3	94.2	93.5	
Yes	3.2	2.7	5.8	6.5	
Sufficiency of received help^a,b,d^					< 0.001
Sufficient	91.6	93.1	89.3	79.0	
In between sufficient/insufficient	5.8	5.5	5.3	14.5	
Insufficient	2.0	1.4	5.3	6.5	
Health problems limit normal activities^a,b^					< 0.001
No	60.8	63.5	48.7	41.9	
Yes, slightly	29.5	28.1	34.4	43.5	
Yes, severely	9.6	8.3	16.9	14.5	
Self-perceived health^b^					< 0.001
Very good to excellent	29.2	31.7	21.4	11.5	
Good	53.4	55.5	50.8	32.8	
Fair to poor	16.1	12.8	27.8	55.7	
*Cognitive vulnerability*					
MMSE score^b^					< 0.001
≥ 27	83.0	83.1	88.9	66.1	
10–26	16.9	16.9	11.1	33.9	
Problems with memory^b^					< 0.001
No	66.0	69.0	53.4	43.5	
Yes	33.8	31.0	46.6	56.5	
*Social vulnerability*					
Loneliness^b^					< 0.001
Not lonely (0–2)	74.2	77.7	65.1	29.0	
Lonely (3–11)	25.8	22.3	34.9	71.0	
Network size^b^					< 0.001
0–12	34.6	33.2	35.7	63.6	
13–20	30.5	31.1	28.6	21.8	
21–70	34.9	35.7	35.7	14.5	
Health problems limit social activities^a,b^					< 0.001
Little/none of the time	76.3	80.6	61.5	39.3	
Some of the time	18.2	15.9	28.3	37.7	
Most/all of the time	5.0	3.4	10.2	23.0	
Self-perceived quality of life^a,b,d^					< 0.001
Rather/very good	84.6	89.1	72.2	35.5	
Neither poor nor good	12.6	9.5	24.1	45.2	
Rather/very poor	2.4	1.5	3.7	19.4	
Financial problems^a,b,d^					< 0.001
No	97.4	98.3	93.9	89.1	
Yes	2.6	1.7	6.1	10.9	
Conflict with other person(s)^b^					< 0.001
No	89.1	90.5	80.7	83.6	
Yes	10.9	9.5	19.3	16.4	
Illness of partner or spouse^a,f^					0.558
No	81.9	82.4	78.2	81.5	
Yes	18.1	17.6	21.8	18.5	
Death of sons, daughters and/or grandchildren^c,d,f^					0.037
No	98.6	98.8	98.7	94.1	
Yes	1.4	1.2	1.3	5.9	
*Psychological vulnerability*					
Depressive symptoms^b^					< 0.001
Normal (< 16)	88.5	92.1	77.2	49.2	
Depressive symptoms (≥ 16)	11.4	7.9	22.8	50.8	
Depression past year^a,b,d^					< 0.001
No depression	96.9	99.1	93.5	82.5	
Depression	2.2	0.9	6.5	17.5	
Anxiety^a,b,d^					< 0.001
Normal (< 7)	92.3	94.8	84.1	65.6	
Suggestive for anxiety disorder (8–10)	4.9	3.5	11.1	16.4	
Anxiety disorder (> 10)	2.7	1.7	4.8	18.0	
Self-esteem^*g*^	15.6 (2.1)	15.3 (3.6)	14.1 (4.2)	11.5 (5.5)	< 0.001
Perceived self-efficacy	44.0 (5.6)	44.3 (5.5)	43.3 (5.6)	40.0 (5.9)	< 0.001
Mastery	24.7 (4.1)	25.1 (4.0)	23.3 (4.2)	21.6 (4.2)	< 0.001
Lately satisfied with life^a,b^					< 0.001
(Very) satisfied	83.7	88.2	70.7	38.7	
Not dissatisfied/satisfied	12.3	9.4	22.3	45.2	
(Very) dissatisfied	3.5	2.4	6.9	16.1	
Meaningfulness of life^a,b^					< 0.001
Very much to an extreme amount	62.4	67.7	43.9	11.3	
A moderate amount	34.0	30.5	48.7	67.7	
A little to not at all	3.2	1.8	7.5	21.0	

n, number of participants; µ, mean; SD, standard deviation; * current wish to die or a weak wish to live

^a^Minimum expected count between 1 and 6

^b^Less than 5% missing values

^c^Minimum expected count less than 1

^d^Fisher–Freeman–Halton Exact Test was used

^e^Chronic diseases: chronic non-specific lung disease, cardiac disease, peripheral arterial disease, diabetes mellitus, cerebrovascular accident or stroke, osteoarthritis and rheumatoid arthritis, cancer

^f^Above 5% missing values; illness of partner/spouse: 28.9% and death of sons, daughters and/or grandchildren: 11.5%

^g^Kruskal–Wallis test was used

### Characteristics associated with a current wish to die

Table 2 shows the results of the univariate analyses. Characteristics for which there was insufficient statistical evidence for an association with the dependent variable (*p* value > 0.05) were: sex, level of education, visual impairment and illness of partner or spouse. In some respects, people who have at some point experienced death thoughts or wishes and people who had a current wish to die were similar. Both groups consisted of relatively many divorced people (13.8–14.5% compared to 6.9%), relatively many people who received personal care (5.8–6.5% compared to 2.7%), and many people who had a conflict with another person (16.4–19.3% compared to 9.5%) in comparison with people who never had death thoughts or wishes. In most factors a gradient was present across the three groups, for example: having a depression (never: 0.9%; at some point: 6.5%; current: 17.5%); having an anxiety disorder (never: 1.7%; at some point: 4.8%; current: 18.0%); and having little/no meaningfulness of life (never: 1.8%; at some point; 7.5%; current: 21.0%). The factors that differed most between the three groups were: loneliness (never: 22.3%; at some point: 34.9%; current: 71%), depressive symptoms according to the CES-D (never: 7.9%; at some point: 22.8%; current: 50.8%), a fair to poor self-perceived health (never: 12.8%; at some point: 27.8%; current: 55.7%), widowhood (never: 16.4%; at some point: 17.5%; current: 35.5%) and an MMSE score between 10 and 26 (never: 16.9%; at some point: 11.1%; current: 33.9%).

In the multivariable analyses, many factors, within all four domains of vulnerability, were related to having a current wish to die (Table 3). In the category of demographics, being divorced [OR 3.55 (1.61–7.84)] and being widowed [OR 2.82 (1.46–5.45)] were associated with having a current wish to die. In the domain of physical vulnerability, self-perceived health was strongly associated with having a current wish to die [OR 9.07 (3.76–21.86)]. Other associated physical characteristics were having much difficulty to hear/not able to hear [OR 2.89 (1.13–7.36)] and feeling dizzy [OR 2.05 (1.04–4.04)]. In the domain of cognitive vulnerability, problems with memory were associated with having a current wish to die [OR 2.82 (1.68–4.75)]. Within the domain of social vulnerability, having a rather/very poor self-perceived quality of life was strongly associated with having a current wish to die [OR 14.15 (4.64–43.12)]. Other associated characteristics within this domain were having a low network size of 0–12 persons [OR 2.45 (1.03–5.81)], feeling lonely [OR 4.65 (2.39–9.04)], and having health problems that limit social activities sometimes [OR 2.21 (1.05–4.69)]. In the domain of psychological vulnerability, having a little to no meaningfulness of life was strongly associated with a current wish to die [OR 24.61 (7.39–81.89)]. Other associated psychological characteristics were having a moderate amount of meaningfulness of life [OR 8.34 (3.36–20.75)], having a major depressive disorder [OR 8.28 (2.63–26.04)], being (very) dissatisfied with life [OR 5.88 (2.11–16.36)], being not dissatisfied/satisfied with life [OR 3.47 (1.69–7.10)] and having a subclinical depression [OR 2.34 (1.12–4.88)].

**Table 3:** Logistic regression analyses of variables associated with having a current wish to die or a weak wish to live compared to has never had death thoughts or wishes, per domain of vulnerability

	Logistic regression analysis n = 1374		
	Odds ratio^a^	95% CI	*p* value
*Demographics*			
Marital status			
Never married	2.149	0.797–5.791	0.131
Married and registered partnership	Ref		
Divorced	3.554	1.612–7.837	0.002
Widowhood	2.819	1.459–5.447	0.002
*Domain 1. Physical vulnerability*			
Hearing impairment			
No impairment	Ref		
Some difficulty	0.953	0.497–1.828	0.884
Much difficulty/not able to hear	2.885	1.131 – 7.356	0.027
Self-perceived health			
Very good to excellent	Ref		
Good	1.440	0.593–3.493	0.421
Fair to poor	9.065	3.759–21.861	< 0.001
Dizziness			
No	Ref		
Yes	2.052	1.044–4.034	0.037
*Domain 2. Cognitive vulnerability*			
Problems with memory			
No	Ref		
Yes	2.821	1.675–4.752	< 0.001
*Domain 3. Social vulnerability*			
Self-perceived quality of life			
Rather/very good	Ref		
Neither poor nor good	5.307	2.539–11.095	< 0.001
Rather/very poor	14.150	4.644–43.118	< 0.001
Network size			
21–70	Ref		
13–20	1.427	0.542–3.757	0.472
0–12	2.450	1.033–5.813	0.042
Loneliness			
Not lonely	Ref		
Lonely	4.646	2.387–9.042	< 0.001
Health problems limit social activities			
Little/none of the time	Ref		
Some of the time	2.214	1.045–4.691	0.038
Most/all of the time	2.186	0.811–5.894	0.122
*Domain 4. Psychological vulnerability*			
Meaningfulness of life			
Very much to an extreme amount	Ref		
A moderate amount	8.344	3.356–20.749	< 0.001
A little to not at all	24.606	7.393–81.893	< 0.001
Lately satisfied with life			
(very) satisfied	Ref		
Not dissatisfied/satisfied	3.466	1.692–7.101	0.001
(very) dissatisfied	5.878	2.112–16.357	0.001
Depression			
No depression	Ref		
Subclinical depression (SUBD)	2.338	1.121–4.876	0.024
Major depressive disorder (MDD)	8.281	2.633–26.044	< 0.001

Housing, and death of sons, daughters and grandchildren were left out of the multivariate analyses because of a minimum expected count less than 1

*n* number of participants, *CI* confidence interval, *Ref* reference

^a^Corrected for age

### Medically classifiable and age-related health problems

Table 4 shows the results of all participants with a current wish to die assessed by three SCEN physicians on having a medically classifiable condition and an accumulation of age-related health problems. The SCEN physicians initially assessed 18 cases differently on having a medically classifiable condition and 18 cases differently on having an accumulation of age-related health problems. Consensus on these cases was reached after discussion, which was related to how to operationalize and classify the two terms regarding the available information (see details in Box 2). Fifty-four of the 62 cases were assessed with having a medically classifiable condition. Of these 54 cases, a majority did not have an accumulation of age-related health problems (n = 47) according to the judgement of the SCEN physicians. Six out of 62 cases (9.7%) neither had a medically classifiable condition nor had an accumulation of age-related health problems according to the judgement of the SCEN physicians.
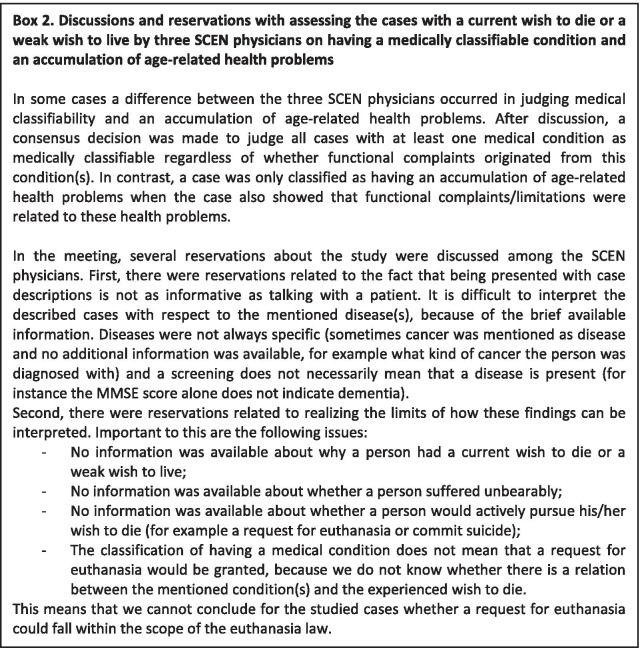

**Table 4:** Assessed cases with a current wish to die or a weak wish to live on having a medically classifiable condition and an accumulation of age-related health problems

	Accumulation of age-related health problems			
	Likely/almost certain	Uncertain	Likely/almost certainly not	Total
Medically classifiable condition				
Likely/almost certain	1 (1.6%)	6 (9.7%)	47 (75.8%)	54 (87.1%)
Uncertain	0 (0.0%)	0 (0.0%)	2 (3.2%)	2 (3.2%)
Likely/almost certainly not	0 (0.0%)	0 (0.0%)	6 (9.7%)	6 (9.7%)
Total	1 (1.6%)	6 (9.7%)	55 (88.7%)	62 (100.0%)

Percentages are of total (n = 62)

## Discussion

We found that 4.0% of study participants had a current wish to die or a weak wish to live (unweighted and weighted), of which 0.7% had a moderate to strong wish to die. A current wish to die was associated with all four domains of vulnerability: physical, cognitive, social and psychological vulnerability. People with a current wish to die had on average more often much difficulty to hear or were not able to hear, had a fair to poor self-perceived health, problems with dizziness and with their memory, a neither poor nor good and a rather/very poor quality of life, a small network size, a subclinical depression and a major depressive disorder, a moderate amount of meaningfulness of life and a little to no meaningfulness of life, and some of the time had limited social activities because of their health problems. Furthermore, people with a current wish to die were on average more often divorced or widowed, lonely, and not dissatisfied/satisfied and (very) dissatisfied with life. Most of the participants with a current wish to die had a medically classifiable condition according to the judgement of the SCEN physicians (n = 54). Furthermore, most of those participants were not classified as having an accumulation of age-related health problems. Of the participants with a current wish to die, six participants did not have a medically classifiable condition, neither did they have an accumulation of age-related health problems according to the judgement of the SCEN physicians.

### Prevalence of a current wish to die

The prevalence of middle-aged and older adults with a current wish to die found in this study (4.0%) is similar to the results of a comparable Dutch study in 2005 of a previous wave of LASA that found a prevalence of 3.4% among Dutch middle-aged and older adults aged between 58 and 98 [6]. If the small rise in prevalence reflects a real change, it might be due to the rising societal discussions about a self-chosen end of life over the last decade. The most recent Dutch study among middle-aged and older adults aged 55 and above found a lower prevalence compared to our study [7], which might be explained by the fact that they excluded participants who are severely ill. Worldwide prevalence estimates of a current wish to die among older adults lay between 4.6 and 9.5% [1–5]. The prevalence in the Netherlands seems lower, although comparing these findings is difficult because of the variation in study population (such as age range) and the method used to measure a current wish to die. In our study, a current wish to die is most common in people aged 85 years or older. Nevertheless, 1 out of 5 participants with a current wish to die are younger than 65 years of age, showing that developing a wish to die is not only limited to older adults, but can also develop in middle-aged people.

### All domains of vulnerability related to a current wish to die

We found many characteristics to be associated with a current wish to die of which eleven remained significantly associated in the multivariable model, spread over all four domains of vulnerability: physical, cognitive, social and psychological vulnerability. The results of our study underscore the multi-faceted nature of current wishes to die, which involve multiple domains of vulnerability. Characteristics such as having a little to no meaningfulness of life, a rather/very poor quality of life, a fair to poor self-perceived health and a major depressive disorder were strongly associated with a current wishes to die. Having a depression and being lonely have been extensively explored and found to be associated with a current wish to die [3, 5–7, 26–28], as well as marital status [6, 8, 26], hearing impairment [6, 28], self-perceived health [4–6, 26], problems with memory [5], network size [6, 26], social activities limited by health problems [5], and lately satisfied with life [26]. It is noticeable that only 17.5% of the participants with a current wish to die were diagnosed with having a depression and 50.8% with having depressive symptoms. This shows that a wish to die and having depressive symptoms or a depression do not always co-exist, which is in line with results from other studies [6, 8]. As far as we know, associations between quality of life, meaningfulness of life and dizziness and a wish to die have not been studied before using quantitative data. However, a qualitative study of Rurup et al. [8] found that a poor quality of life is one of five triggers of the development of a wish to die. Related to meaningfulness of life, this study and two other qualitative studies also found that feeling redundant, no longer useful or no longer important are factors that could lead to a wish to die [8, 10, 11]. In addition, people with a wish to die often mentioned feeling life had no meaning anymore [11, 29].

### Current Dutch political debate

In the ongoing debate in the Netherlands about whether or not a new law has to be introduced for people who experience a completed life [17], Committee Schnabel expected the group of older adults who experience a completed life without having a medically classifiable condition to be small [17]. They assumed that most older adults who experience a completed life have an accumulation of age-related health problems and fall within the scope of the current euthanasia Act [17]. However, their assumptions were not based on empirical findings, because studies on this issue were lacking, until then [17]. We found that the group of middle-aged and older adults with a current wish to die is indeed small. However, in contrast to what Committee Schnabel assumed, it seems that a majority of middle-aged and older adults with a current wish to die who have a medically classifiable condition do not seem to have an accumulation of age-related health problems. In interpreting these results, it is important to consider the reservations that were stressed by the SCEN physicians. It is especially important to realize that for people with a current wish to die who were assessed with having a medically classifiable disease, we do not know whether their wish to die is related to their disease. This is necessary to assess whether or not a request for euthanasia might fall within the scope of the euthanasia Act. Furthermore, we do not know whether the participants with a current wish to die in this study actively would want to pursue ending their life, for example by requesting euthanasia.

### Strengths and limitations

Major strengths of this study are the broad range of domains for which measurements were available within LASA, and the fact that three SCEN physicians, who are experts in this field, assessed whether or not the participants with a current wish to die had a medically classifiable condition and an accumulation of age-related health problems. However, this study also has limitations. The group with a current wish to die was relatively small, therefore the associations have to be interpreted with some caution. Still, some results in this study were very pronounced and some associations could be demonstrated with sufficient statistical evidence. Another limitation is the fact that three somewhat different groups were merged into one group with a current wish to die: those who had only a weak wish to live in the past week, but no wish to die in the past week; those who had no wish to live in the past week and/or a weak wish to die in the past week; and those who had a moderate to strong wish to die in the past week. In some respects, these groups noticeably differed in characteristics. People with a moderate to strong wish to die had more often depressive symptoms, MMSE score between 10 and 26, much difficulty to hear or were not able to hear, were more often lonely and diagnosed with a depression compared to the other two groups. This could have led to underestimated effects. However, research shows that a wish to die and a wish to live can occur together [8, 9]. Furthermore, it is possible that due to our broad age range observed associations might have been different when focusing on a narrower age group. Finally, cases with a current wish to die assessed by the SCEN physicians were described with available data from LASA, which in some respects was brief. In the case descriptions we mentioned a person’s physical and mental health, however, we had no data whether a person’s wish to die was related to his/her medical condition and if their medical condition caused unbearable suffering. Reservations regarding this are described in box 2.

## Conclusion

In conclusion, a small minority of middle-aged and older adults in the Netherlands have a current wish to die or a weak wish to live. Multiple factors, within all four domains of vulnerability, are associated with a current wish to die. Most participants with a current wish to die can be classified with a medical condition and one person with an accumulation of age-related health problems. Furthermore, the findings show that it is important to realize that having a current wish to die among middle-aged and older adults is multi-faceted, including not only social vulnerability but also a broader range of vulnerability. This should be considered in the prevention of developing a wish to die. However, there is still a need for more knowledge, such as insight in to what extent suffering stemming from the medical classifiable disease contributes to the development of the wish to die, how many people with a wish to die actively would want to pursue their wish to die, and insight in the persistency of wishes to die.

## Supplementary Information

**Additional file 1.** Title and description of data: Recoding of the four questions into the dependent variable. **Additional file 2.** Title and description of data: Independent variables and corresponding recodes. **Additional file 3.** Title and description of data: Example of a fictitious case description of a respondent with a current wish to die.

## Data Availability

The data that support the findings of this study are available from the Longitudinal Ageing Study Amsterdam but restrictions apply to the availability of these data, which were used under license for the current study, and so are not publicly available. Data are however available from the authors upon reasonable request and with permission of the Longitudinal Ageing Study Amsterdam (www.lasa-vu.nl).

## Abbreviations

CI: Confidence interval
EAS: Euthanasia and physician-assisted suicide
LASA: Longitudinal Aging Study Amsterdam
OR: Odds ratio
SCEN: Support and Consultation on Euthanasia in the Netherlands
SD: Standard deviation
