# Supplementary material for: Current wishes to die; characteristics of middle-aged and older Dutch adults who are ready to give up on life: a cross-sectional study
Source: BMC Med Ethics. 2021 May 21;22:64. doi: 10.1186/s12910-021-00632-4 (PMC8140496; doi:10.1186/s12910-021-00632-4)
Supplement: Supplementary file 1 — Additional file 1. Title and description of data: Recoding of the four questions into the dependent variable. Additional file 2. Title and description of data: Independent variables and corresponding recodes. Additional file 3. Title and description of data: Example of a fictitious case description of a respondent with a current wish to die. [file 12910_2021_632_MOESM1_ESM.docx]

Current wishes to die; characteristics of middle-aged and older Dutch adults who are ready to give up on life: a cross-sectional study

Roosmarijne M.K. Kox*^1^, H. Roeline W. Pasman^1^, Martijn Huisman^23^, Wim Benneker^4^ and Bregje D. Onwuteaka-Philipsen^1^

* Correspondence: [r.kox@amsterdamumc.nl](mailto:r.kox@amsterdamumc.nl)

^1^Amsterdam UMC, University of Amsterdam, Department of Public and Occupational Health, Amsterdam Public Health research institute, P.O. Box 7057, 1007 Amsterdam, MB, Netherlands.

^2^Department of Epidemiology and Biostatistics, Amsterdam Public Health Research Institute, Amsterdam UMC - Location VU University Medical Center, Amsterdam, The Netherlands.

^3^Department of Sociology, Vrije Universiteit Amsterdam, Amsterdam, The Netherlands.

^4^Department of General Practice and Elderly Care Medicine, University of Groningen, University Medical Center Groningen.

**APPENDIX**

**Additional file 1. Recoding of the four questions into the dependent variable**

| **Variable** | **Question / item(s)** | **Response categories** | **Recodes** | |
| --- | --- | --- | --- | --- |
| Ever having death thoughts or wishes | - ‘Have you ever felt like life was not worth living?’ - ‘Have you ever wished you were dead, for instance, that you could go to sleep and not wake up?’ | 1. Yes 2. No 3. Don’t know | Recoded into new variable:   1. Has never had death thoughts or wishes   *(= ‘no’ or ‘don’t know’ on both questions about ever had death thoughts or wishes)*   1. Has at some point experienced death thoughts and/or wishes, but had no wish to die in the past week and a moderate to strong wish to live in the past week   *(= ‘yes’ on at least one question about ever had death thoughts or wishes)*   1. Had only a weak wish to live in the past week, but no wish to die in the past week   *(= ‘a weak wish to live’ on question about feelings toward living and ‘no wish to die’ on question about feelings toward dying)*   1. Had no wish to live in the past week and/or a weak wish to die in the past week   *(= ‘no wish to live’ on question about feelings toward living and/or ‘a weak wish to die’ on question about feelings toward dying)*   1. Had a moderate to strong wish to die in the past week   *(= ‘a moderate to strong wish to die’ on question about feelings toward dying)* | Categorized into:   1. Has never had death thoughts or wishes *(= 1)* 2. Has at some point experienced death thoughts or wishes *(= 2)* 3. Current wish to die or a weak wish to live *(= 3, 4, 5)* |
| Having death wishes in the past week | - ‘What were your feelings toward living the past week? Did you wish to live, and how strong was this wish? Did you have …’ | 1. A moderate to strong wish to live 2. A weak wish to live 3. No wish to live |  |  |
|  | - ‘What were your feelings toward dying the past week? Did you wish to die, and how strong was this wish? Did you have …’ | 1. No wish to die 2. A weak wish to die 3. A moderate to strong wish to die |  |  |

**Additional file 2. Independent variables and corresponding recodes**

| **Variable** | **Question / item(s)** | **Answer categories** | **Recodes** | | | |
| --- | --- | --- | --- | --- | --- | --- |
| **Demographics** |  | | | | | |
| Sex | Sex of the participant | 1. Male 2. Female | n.a. | | | |
| Age | Age of the participant | Continuous | Categorized into:   1. < 65 2. 65 - <75 3. 75 - <85 4. ≥ 85 | | | |
| Level of attained education | Highest attained level of education | 1. Elementary not completed 2. Elementary education 3. Lower vocational education 4. General intermediate education 5. Intermediate vocational education 6. General secondary education 7. Higher vocational education 8. College education 9. University education | Categorized into:   1. Low (*=categories 1-3*) 2. Middle (*=categories 4-6*) 3. High (*=categories 7-9*) | | | |
| Marital status | ‘What is your official marital status?’ | 1. Never married 2. Married 3. Divorced 4. Widowhood 5. Registered partnership | Categorized into:   1. Never married 2. Married & registered partnership 3. Divorced 4. Widowhood | | | |
| Level of urbanization | The level of urbanization is the mean number of addresses per squared kilometer within a circle with a radius of one kilometer. | 1. Not (<500) 2. Little (500-1000) 3. Somewhat (1000-1500) 4. Highly (1500-2500) 5. Very highly (≥2500) | Dichotomized into:   1. Sparsely populated *(= <1,000)* 2. Densely populated *(= ≥1,000)* | | | |
| Housing | Type of housing was observed | 1. Independent 2. Residential home 3. Nursing home - somatic | Dichotomized into:   1. Dependent (residential or nursing home) 2. Independent | | | |
| **Physical vulnerability** |  | | | | | |
| Number of chronic diseases (out of 7) | The participants were asked about the following seven most frequently occurring chronic diseases in the Netherlands:   - chronic non-specific lung disease - cardiac disease - peripheral arterial disease - diabetes mellitus - cerebrovascular accident or stroke - osteoarthritis and rheumatoid arthritis - cancer | Continuous | Categorized into:   1. 0 2. 1 3. ≥ 2 | | | |
| Incontinence | ‘Did you have had unintentional urine loss in the last month due to a strong urge and not being able to make it to the toilet in time?’ | 1. 2 times a month or less 2. 3-4 times a month 3. A few times a week 4. Daily | Dichotomized into:   1. No *(=categories 1 and 2)* 2. Yes *(=categories 3 and 4)* | | | |
| Pain | A pain scale was used (based on a subscale from the Nottingham Health Profile)(1). | Continuous (sum score) | Categorized (2) into:   1. No pain *(= 5)* 2. Some pain *(= 6-7)* 3. Many pain *(= >7)* | | | |
| Hearing impairment | ‘Can you hear well enough?’ (Regardless of using a hearing aid) | 1. Yes, without difficulty 2. Yes, with some difficulty 3. Yes, with much difficulty 4. No, I cannot | Categorized into:   1. No impairment *(=category 1)* 2. Some difficulty *(=category 2)* 3. Much difficulty/not able to hear *(=categories 3 and 4)* | | | |
| Visual impairment | - ‘Are you able to read the normal, lowercase letters in the newspaper without glasses/lenses?’ | 1. Yes, without difficulty 2. Yes, with some difficulty 3. Yes, with much difficulty 4. No, I cannot | Recoded into new variable (vision nearby):   1. No impairment   *(=‘yes, without difficulty’ on question 1; and ‘yes, without difficulty’ on question 2 and ‘yes, with some difficulty’ or worse on question 1)*   1. Some difficulty   *(=‘yes, with some difficulty’ on question 1 and ‘yes,* *with some difficulty’ or worse on question 2, and the other way around)*   1. Much difficulty/not able to see *(=‘yes, with much difficulty’ or ‘no, I cannot’ on question 1 and ‘yes, with some difficulty’ or worse on question 2 or the other way around)* | | Categorized into:   1. No impairment   *(=‘no impairment’ on both vision nearby and on distance)*   1. Some difficulty   *(=‘some difficulty’ on both variables; and ‘some difficulty’ on vision nearby and ‘no impairment’ on vision from distance, or the other way around)*   1. Much difficulty/not able to see *(=‘much difficulty/not able to see’ on both variables; and ‘some difficulty’ on vision nearby and ‘much difficulty/not able to see’ on vision from distance, and the other way around; and ‘no impairment’ on vision nearby and ‘much difficulty/not able to see’ on vision from distance, and the other way around)* | |
|  | - ‘Are you able to read the normal, lowercase letters in the newspaper with glasses/lenses?’ | 1. Yes, without difficulty 2. Yes, with some difficulty 3. Yes, with much difficulty 4. No, I cannot 5. Participant does not have glasses or contact lenses |  |  |  |  |
|  | - ‘Are you able to recognize someone’s face at a distance of 4 meters without glasses/lenses?’ | 1. Yes, without difficulty 2. Yes, with some difficulty 3. Yes, with much difficulty 4. No, I cannot | Recoded into new variable (vision on distance):   1. No impairment   *(=‘yes, without difficulty’ on question 3; and ‘yes, without difficulty’ on question 4 and ‘yes, with some difficulty’ or worse on question 3)*   1. Some difficulty   *(=‘yes, with some difficulty’ on question 3 and ‘yes, with some difficulty’ or worse on question 4, and the other way around)*   1. Much difficulty/not able to see *(=‘yes, with much difficulty’ or ‘no,*   *I cannot’ on question 3 and ‘yes, with some difficulty’ or worse on question 4 or the other way around)* | |  |  |
|  | - ‘Are you able to recognize someone’s face at a distance of 4 meters with glasses/lenses?’ | 1. Yes, without difficulty 2. Yes, with some difficulty 3. Yes, with much difficulty 4. No, I cannot 5. Participant does not have glasses or contact lenses |  |  |  |  |
| Dizziness | ‘Do you feel dizzy regularly?’ | 1. No 2. Yes | n.a. | | | |
| Number of activities with some difficulty or worse | - ‘Can you walk up and down a staircase of 15 steps without resting?’ | 1. No, I cannot 2. Only with help 3. Yes, with much difficulty 4. Yes, with some difficulty 5. Yes, without help | Recoded into seven new variables (per question):   1. No difficulty (=category 5) 2. Some difficulty or worse (=categories 1 to 4) | Recoded into one new variable (2):  Continuous (sum score) | | Categorized into:   1. 0 activities 2. 1 activity 3. ≥ 2 activities |
|  | - ‘Can you dress and undress yourself?’ |  |  |  |  |  |
|  | - ‘Can you sit down and stand up from a chair?’ |  |  |  |  |  |
|  | - ‘Can you cut your own toenails?’ |  |  |  |  |  |
|  | - ‘Can you walk outside during five minutes without stopping?’ |  |  |  |  |  |
|  | - ‘Can you use your own or public transportation?’ |  |  |  |  |  |
|  | - ‘Can you take a shower or bath?’ |  |  |  |  |  |
| Balance | During the test, respondents had to keep both feet close behind each other, so that the heel of one foot and the toes of the other foot touched (tandem position); they had to keep this up for 30 seconds. | 1. Normal test 2. Not capable 3. Falls almost direct 4. Refusal 5. Physically impossible (for example cannot stand on both legs) | Categorized into:   1. No balance disorder (*=category 1*) 2. Balance disorder (*=categories 2 and 3*) 3. Refused/not able to (*=categories 4 and 5*) | | | |
| Whether or not help was received with personal care | ‘Do you receive personal care?’ | 1. No 2. Yes | n.a. | | | |
| Sufficiency of received help | ‘Is the help you receive sufficient?’ | 1. Sufficient 2. In between sufficient/insufficient 3. Insufficient | n.a. | | | |
| Health problems limit normal activities | ‘Do you feel limited in your normal activities because of your health problems?’ | 1. No 2. Yes, slightly 3. Yes, severely | n.a. | | | |
| Self-perceived health | Participants were asked about their self-perceived health. | 1. Excellent 2. Very good 3. Good 4. Fair 5. Poor | Categorized into:   1. Very good to excellent 2. Good 3. Fair to poor | | | |
| **Cognitive vulnerability** |  | | | | | |
| MMSE score | The Mini-Mental State Examination (MMSE) was used, which assesses the cognitive functioning (3). The higher the score, the better the cognitive performance. | Continuous | Dichotomized (4) into:   1. *>26* 2. *10–26* | | | |
| Problems with memory | ‘Do you experience problems with your memory?’ | 1. No 2. Yes | n.a. | | | |
| **Social vulnerability** |  | | | | | |
| Loneliness | The 11-item scale of De Jong Gierveld (5) was used. | Continuous (sum score) | Dichotomized (6) into:   1. Not *lonely (= 0–2)* 2. Lonely *(= 3–11)* | | | |
| Network size | Network size was measured by asking the participants about their socially active relationships. A total network size of 80 was the limit, nominating only people above the age of 18. | Continuous | Categorized into:   1. 0 – 12 2. 13 – 20 3. 21 – 70 | | | |
| Health problems limit social activities | ‘During the past 4 weeks, how much of the time has your physical health or emotional problems interfered with your social activities (like visiting with friends, relatives, etc.)?’ | 1. All of the time 2. Most of the time 3. Some of the time 4. A little of the time 5. None of the time | Categorized into:   1. Most/all of the time 2. Some of the time 3. Little/none of the time | | | |
| Self-perceived quality of life | Participants were asked about their self-perceived quality of life. | 1. Very poor 2. Rather poor 3. Neither poor nor good 4. Rather good 5. Very good | Categorized into:   1. Rather/very poor 2. Neither poor nor good 3. Rather/very good | | | |
| Financial problems | ‘Did you have had severe financial problems in the past three years?’ | 1. No 2. Yes | n.a. | | | |
| Conflict with other person(s) | ‘Did you have had a conflict with another person(s) in the past three years?’ | 1. No 2. Yes | n.a. | | | |
| Illness of partner or spouse | ‘Did your partner/spouse had a severe illness in the past three years?’ | 1. No 2. Yes | n.a. | | | |
| Death of sons, daughters and/or grandchildren | - ‘Did you have a son(s) that passed away in the past three years?’ | 1. No 2. Yes | Categorized into:   1. No   *(=‘no’ or ‘missing’ on all three questions)*   1. Yes   *(=’yes’ on at least one question)* | | | |
|  | - ‘Did you have a daughter(s) that passed away in the past three years?’ |  |  |  |  |  |
|  | - ‘Did you have a grandchild(ren) that passed away in the past three years?’ |  |  |  |  |  |
| **Psychological vulnerability** |  | | | | | |
| Depressive symptoms | The Self-report Center for Epidemiologic Studies Depression Scale (CES-D) (7) was used. | Continuous | Dichotomized (2) into:   1. Normal *(= <16)* 2. Clinically relevant depressive symptoms *(= ≥16)* | | | |
| Depression past year | Respondents with a HADS-A score equal to or above 8, and/or a CES-D score equal to or above 16 were screened for a depressive disorder (in the past year) with the Composite International Diagnostic Interview (CIDI). | 1. Negative 2. Positive | 1. No depression 2. Depression | | | |
| Anxiety | Anxiety subscale of the Hospital Anxiety Depression Scale (HADS-A) (8) was used | Continuous | Categorized (2) into:   1. Normal *(=score of 0 to 7)* 2. Suggestive for an anxiety disorder *(=score of 8 to 10)* 3. Presence of an anxiety disorder *(=score of 11 and higher)* | | | |
| Self-esteem | An adapted version of Rosenberg Self-esteem scale (9) was used, which consisted of 4 out of 10 items. | Continuous | n.a. | | | |
| Perceived self-efficacy | A 12-item version (10) of the General Self-Efficacy Scale (11) (GSES-12) was used, resulting in a score between 12 and 60. | Continuous | n.a. | | | |
| Mastery | The Pearlin Mastery Scale (12) was used | Continuous | n.a. | | | |
| Lately satisfied with life | Respondents were asked about their lately satisfaction with life. | 1. Very dissatisfied 2. Dissatisfied 3. Not dissatisfied/satisfied 4. Satisfied 5. Very satisfied | Categorized into:   1. (very) Dissatisfied 2. Not dissatisfied/satisfied 3. (very) Satisfied | | | |
| Meaningfulness of life | Respondents were asked about their meaningfulness of life. | 1. Not at all 2. A little 3. A moderate amount 4. Very much 5. An extreme amount | Categorized into:   1. A little to not at all 2. A moderate amount 3. Very much to an extreme amount | | | |

**Additional file 3. Example of a fictitious case description of a respondent with a current wish to die**

Case 1.

**Background**

This case is about a [male/female] with an age between 85 and 95 years old.

**Diseases**

*Chronic diseases / incontinence*

Of all chronic diseases asked, [Participant] has:

- one heart attack in the last three years
- peripheral arterial disease in stomach or legs
- osteoarthritis
- prostate cancer and cancer in vertebra
- bone metastases
- different: back problems, hernia

[Participant] has had two times a month or less a strong urge to urinate and unable to make it to the toilet on time in the past month

*Dementia / cognitial problems*

[Participant] has an MMSE score of 27 (no dementia) and [participant] experiences no problems with own memory.

*Depressive symptoms / depression / anxiety*

[Particpant] has a CES-D score of 14 (no depressive symptoms), no depression was diagnosed in the past month or past year (according to CIDI score). [Participant] has an HADS-A anxiety score of 9 (suggestive for anxiety).

*Pain*

Of the six statements about pain, [participant] answered with ‘yes’ on: I am in pain when I am standing; I find it painful to change position; I am in pain when I walk; I am in constant pain.

*Dizziness*

[Participant] answered with ‘no’ on the question if he/she is regularly dizzy.

**Functional well-being**

*Hearing and visual perception*

[Participant] is able to hear without difficulty, without using a hearing aid. [Participant] is wearing glasses/contact lenses. [Participant] is without difficulty able to read the normal, small print in the newspaper without glasses/contact lenses. [Participant] is without difficulty able to recognize someone’s face from a distance of 4 meters without glasses or contact lenses.

*Balance*

[Participant] has a balance disorder, he/she fell almost directly after starting the test.

*ADL activities*

Of the seven ADL activities that have been asked, [participant] is not able to use own or public transport. [Participant] is able to do the other six activities without help.

**Self-perceived health**

[Participant] perceives his/her health as ‘fair’; [Participant] perceives his/her health from three years ago as ‘excellent’.

[Participant] experiences that his/her health problems do not limit normal activities and [participant] experiences that social activities are some of the time limited by his/her health or emotional problems.

According to your judgement, does this person have one (or more) medically classifiable condition(s)?

- Almost certain
- Likely
- Uncertain
- Likely not
- Almost certainly not

Explanation (especially when in doubt):

……………………………………………………………………………………………………………………………………………………………………………………………………………………………………………………………………………………………………………………………………………………………………………………………………………………………………………………………………………………………………………………………………………………………………………………………………………………………………………………

According to your judgement, does this person have an accumulation of age-related health problems?

- Almost certain
- Likely
- Uncertain
- Likely not
- Almost certainly not

Explanation (especially when in doubt):

……………………………………………………………………………………………………………………………………………………………………………………………………………………………………………………………………………………………………………………………………………………………………………………………………………………………………………………………………………………………………………………………………………………………………………………………………………………………………………………

**REFERENCES**

1. Hunt SM, McEwen J, McKenna SP. Measuring health status: a new tool for clinicians and epidemiologists. The Journal of the Royal College of General Practitioners. 1985;35(273):185-8.

2. Longitudinal Aging Study Amsterdam (LASA). Longitudinal Aging Study Amsterdam [cited 2020 July]. Available from: <https://www.lasa-vu.nl>.

3. Folstein M, Folstein S, McHugh P. Mini-mental state: a practical method for the clinician. Journal of Psychiatric Research. 1975;12:189-98.

4. van Charante EM, Perry M, Vernooij-Dassen M, Boswijk D, Stoffels J, Achthoven L, et al. NHG-Standaard Dementie (derde herziening). Huisarts Wet. 2012;55(7):306-17.

5. De Jong-Gierveld J, Kamphuls F. The development of a Rasch-type loneliness scale. Applied psychological measurement. 1985;9(3):289-99.

6. Van Tilburg T, De Jong-Gierveld J. Cesuurbepaling van de eenzaamheidsschaal. Tijdschrift voor Gerontologie en Geriatrie. 1999;30:158-63.

7. Radloff LS. The CES-D scale: A self-report depression scale for research in the general population. Applied Psychological Measurement. 1977;1(3):385-401.

8. Zigmond AS, Snaith RP. The hospital anxiety and depression scale. Acta Psychiatrica Scandinavica. 1983;67(6):361-70.

9. Rosenberg M. Society and the adolescent self-image. Princeton: Princeton University Press; 1965.

10. Bosscher R, Laurijssen L, De Boer E. Competentie op latere leeftijd: een exploratieve studie. Bewegen & Hulpverlening. 1992;9:255-65.

11. Sherer M, Maddux JE, Mercandante B, Prentice-Dunn S, Jacobs B, Rogers RW. The self-efficacy scale: Construction and validation. Psychological Reports. 1982;51(2):663-71.

12. Pearlin LI, Schooler C. The structure of coping. Journal of Health and Social Behavior. 1978;19:2-21.
